# Genomically Driven Precision Medicine to Improve Outcomes in Anaplastic Thyroid Cancer

**DOI:** 10.1155/2014/936285

**Published:** 2014-09-07

**Authors:** Nicole Pinto, Morgan Black, Krupal Patel, John Yoo, Joe S. Mymryk, John W. Barrett, Anthony C. Nichols

**Affiliations:** ^1^Department of Otolaryngology, Head & Neck Surgery, The University of Western Ontario, London, ON, Canada N6A 3K7; ^2^London Regional Cancer Program, London, ON, Canada N6C 2R6; ^3^Department of Oncology, The University of Western Ontario, London, ON, Canada N6A 3K7; ^4^Lawson Health Research Institute, London, ON, Canada N6C 2R5; ^5^Department of Microbiology and Immunology, The University of Western Ontario, London, ON, Canada N6A 3K7

## Abstract

Thyroid cancer is an endocrine malignancy with an incidence rate that has been increasing steadily over the past 30 years. While well-differentiated subtypes have a favorable prognosis when treated with surgical resection and radioiodine, undifferentiated subtypes, such as anaplastic thyroid cancer (ATC), are far more aggressive and have a poor prognosis. Conventional therapies (surgical resection, radiation, chemotherapy, and radioiodine) have been utilized for treatment of ATC, yet these treatments have not significantly improved the overall mortality rate. As cancer is a genetic disease, genetic alterations such as mutations, fusions, activation of oncogenes, and silencing of tumor suppressors contribute to its aggressiveness. With the use of next-generation sequencing and the Cancer Genome Atlas, mutation-directed therapy is recognized as the upcoming standard of care. In this review, we highlight the known genetic landscape of ATC and the need for a comprehensive genetic characterization of this disease in order to identify additional therapeutic targets to improve patient outcomes.

## 1. Introduction

Cancers of the thyroid are common. Approximately 37,000 new cases of thyroid cancer are diagnosed each year in North America and the incidence is increasing [1]. These types of cancers can be classified into (1) those arising from follicular cells and (2) medullary thyroid cancer arising from calcitonin-producing C cells [2]. The majority of these cancers are well differentiated including papillary thyroid cancers (PTC; overall frequency of 86%) and follicular thyroid cancers (FTC; 9%), with a much lower frequency of poorly differentiated and undifferentiated (anaplastic) thyroid cancers (<2% and 1-2%, resp.) [3, 4]. While most cases of thyroid cancer of follicular cell origin are sporadic, approximately 3.5–6.2% have familial origins, whereby an individual's risk of developing thyroid cancer is >95% if there are three or more affected family members [2]. Whether from a familial origin or sporadic, these types of thyroid cancers are histologically indistinguishable [2]. Well-differentiated cancers typically have an excellent prognosis, with cure rates greater than 90% when treated surgically with or without postoperative radioiodine [5].

In stark contrast, anaplastic (undifferentiated) thyroid cancer (ATC) is rare, accounting for only 2% of thyroid cancers but represents perhaps the most lethal human malignancy, because it is almost universally fatal. The presentation of ATC is often dramatic with a rapidly expanding neck mass leading to airway distress and esophageal obstruction [6, 7]. Distant metastases at presentation are also extremely common (50%), contributing to survival times measured in months and sometimes as little as weeks. Indeed, the median survival in most studies is approximately 6 months following diagnosis, with only 10% of patients surviving at 1 year [6–9]. Thus, there is an urgent need for better treatment options to improve the dismal outcomes of this aggressive cancer.

## 2. Conventional Therapies for Anaplastic Thyroid Cancer

The American Joint Committee on Cancer has grouped ATC into three different stages: stage IVA is characterized by intrathyroidal tumors, stage IVB is characterized by a primary tumor exhibiting extrathyroidal extension, and stage IVC is characterized by distant metastases [9]. In the absence of distant metastases, primary surgery may be a management option, particularly if the disease is completely intrathyroidal (IVA) [10]. If a complete resection is possible, followed by adjuvant therapy, a cure may be possible [10]. However, the majority of tumors present in stage IVB with significant extrathyroidal spread, often involving structures considered unresectable, including the great vessels and prevertebral fascia [10].

ATC is typically considered highly radioresistant; however there are reports of responses to radiation alone, particularly when it is delivered using hyperfractionated techniques [7, 11]. However, even if tumors respond, the effect on long-term survival is minimal. Radiation appears to have the greatest benefit in the postoperative setting, as patients receiving a total or partial thyroidectomy plus postoperative radiotherapy experienced significantly better survival rates than those only biopsied followed by radiotherapy [7].

While radioactive iodine (RAI) is a cornerstone of treatment for well-differentiated thyroid cancers that are generally iodine avid, ATC is almost universally iodine negative due to its undifferentiated nature. Thus, RAI plays little to no role in the management of ATC.

Chemotherapy has been used concurrently with radiotherapy, or alone in the palliative setting. Unfortunately, control of ATC with single agents or with combinations of chemotherapy drugs (e.g., doxorubicin, etoposide, cisplatin, bleomycin, and vincristine) has not been successful, resulting in low response rates and high toxicity. Patients with undifferentiated thyroid carcinoma given doxorubicin monotherapy exhibited progression of the disease [12]. Using a combination of therapy (e.g., doxorubicin with cisplatin) is superior to using a single-agent, with an observed 30% response in a small patient population [7].

While many studies have reported that chemotherapy alone does not improve survival [13], chemotherapies have been implicated as beneficial when used in multimodal therapies [7]. In some patients, the combination of surgery, chemotherapy, and radiotherapy has been shown to locally control the disease in a subset of cases; however, overall patient survival rates remain extremely poor [7]. Alternate approaches are necessary to improve patient outcomes.

One of the most promising and current anticancer strategies that can be applied to ATC is to target “actionable” mutations identified in tumors, particularly activating mutations, gene fusions, and amplifications affecting oncogenes. The potential of targeted drug therapy was exhibited in one particular case study with a 79-year-old male diagnosed with ATC. Sunitinib, a multitargeted inhibitor of multiple tyrosine kinase receptors, was administered and the patient exhibited a rapid complete response [14]. Further studies of targeted agents paired with genomic characterization of the tumors may provide additional insights in to which populations of patients benefit the most from each agent.

## 3. Next-Generation Sequencing and Thyroid Cancer

Cancer is a genetic disease, and the advent of next-generation sequencing (NGS) has already transformed cancer care. Through large collaborative efforts such as the Cancer Genome Atlas (TCGA), the genetic changes underpinning most common cancers have been mapped out [15]. While the majority of genetic changes represent inactivating mutations in tumor suppressor genes and are not actionable or nonconsequential passenger mutations, a subset of activating mutations in oncogenes appears to be druggable and predict response to targeted molecular agents [15]. Indeed mutation directed therapy is already the standard of care for* BRAF* mutant melanoma,* EGFR* mutant lung cancer, and* KRAS* mutant colorectal cancer, amongst others [16–18].

While a comprehensive genetic analysis of ATC has not been carried out using NGS, the TCGA study of PTC is nearing publication. All data is fully available through http://cBioPortal.org. A current model of thyroid neoplasms suggests that ATC arises from preexisting well-differentiated thyroid carcinoma with additional genetic events [19]. Demeter et al. found that 76% of those diagnosed with ATC had previous or concurrent thyroid disorders with 47% related to well-differentiated thyroid cancers, leading further credence to sequential progression from well-differentiated thyroid cancer to undifferentiated ATC [20]. Unlike many other cancers, the PTC TCGA data reveals few mutations in tumor suppressors such as* TP53*, but a very high rate of activating mutations in oncogenes. These are mutually exclusive, nonoverlapping mutations and/or fusions that occur in* BRAF* (62%),* RAS* (*HRAS* 4%,* NRAS* 8%, and* KRAS* 1%),* ALK* (1%),* RET* (6%), and* NTRK3* (2%) [4, 21–23]. Similarly, follicular and Hürthle cell thyroid carcinomas are dominated by activating mutations in* RAS* (40–50%),* PIK3CA* (~10%), and* PAX8/PPAR* *γ* fusions [4, 24]. Not surprisingly, the above genomic changes observed in well-differentiated thyroid cancers have also been observed in ATC, which is discussed further below [4].

## 4. The Genetic Landscape of Anaplastic Thyroid Cancer

### 4.1. Oncogenes

BRAF is a serine-threonine kinase belonging to the family of RAF proteins. Mutations of* BRAF* occur in 20–40% of ATC cases (Table 1), with almost all involving a thymine to adenine (T > A) transversion at position 1799, resulting in a nonsynonymous substitution of valine-to-glutamate at the 600th residue (V600E) [4]. The V600E* BRAF* mutation results in constitutive activity of the BRAF kinase, which phosphorylates and activates MEK and in turn activates ERK to produce constitutive MAPK pathway stimulation [4, 25–27]. This signal transduction cascade normally couples signals from cell surface receptors to regulate gene expression, influencing proliferation, differentiation, apoptosis, and survival [28, 29]. This mutation has been shown to be associated with aggressive pathological traits, resistance to treatment, and increased recurrence rates [25].

Most importantly, activating mutations in* BRAF* appear to predict sensitivity to BRAF inhibitors and this is perhaps the strongest correlation of drug response with a genetic change in cancer care [30, 31]. Indeed, a dramatic response to BRAF inhibitors in ATC has been reported. A 51-year-old man treated with vemurafenib for* BRAF* V600E mutation positive ATC had complete resolution of the lung metastatic disease [26]. Based on this result, it has been suggested that all ATC patients should be tested for V600E and that BRAF inhibitors should be considered in confirmed positive cases and as empirical treatment in rapidly progressive cases pending results of mutation analysis [26].

*H-, K-*, and* N-RAS* genes encode for GTPase-proteins at the inner cell membrane responsible for activating the MAPK and PI3K/Akt/mTOR pathways to influence cellular proliferation, differentiation, and survival [32]. RAS proteins function as molecular switches. When bound to guanosine diphosphate (GDP) they are inactive. However, when GDP is released and exchanged for guanosine triphosphate (GTP), they become activated and remain active until they hydrolyze GTP to GDP [32]. Point mutations that activate RAS are commonly observed in many cancers, such as tumors of the head and neck [32]. As for many other cancer types,* RAS* mutations associated with thyroid cancers primarily occur in codons 12, 13, and 61 [27]. The first two cause increased affinity for GTP, while the third results in inactivation of GTPase function. These mutations in* RAS* genes (which occur in about 20–40% of ATC cases) (Table 1) result in a constitutively activated protein, which activates signaling pathways downstream [4, 27]. The prevalence of* RAS* mutations in ATC suggests that this could play a role in tumor dedifferentiation. Small molecule RAS inhibitors have been difficult to utilize due to their inability to outcompete its very high affinity for cytosolic GTP and a lack of suitable binding areas on the protein that will allow disruption of RAS effector interactions [33]; however, investigation into directly targetable upstream and downstream pathway members, such as the mitogen-activated protein kinase, is being studied [34, 35].

*CTNNB1* encodes *β*-catenin, a key downstream component of the canonical Wnt signaling pathway, which regulates cellular processes involved in development, differentiation, and tissue homeostasis [32, 36]. A point mutation in exon 3 of* CTNNB1* affects about 5–60% of ATC patients (Table 1). This mutation is prominent in undifferentiated cancers (e.g., 0–25% of poorly differentiated thyroid carcinomas and 66% of undifferentiated thyroid carcinomas) as opposed to other well-differentiated thyroid cancer types, suggesting that this particular mutation contributes to the progression of poorly or undifferentiated thyroid carcinomas [9, 37, 38]. When* CTNNB1* is mutated, *β*-catenin, which is involved in intracellular signaling, becomes constitutively activated via Wnt signaling. Upregulated *β*-catenin translocates to the nucleus and can activate expression of tumor-promoting genes with concomitant mutations in other genes such as* KRAS* and* TP53* [39, 40]. For this reason, efforts are being made to target and inhibit the aberrant Wnt/*β*-catenin signaling pathway. The use of regular nonsteroidal anti-inflammatory drugs (NSAIDS) has been shown to reduce incidence of various types of human cancers. The antitumor actions exhibited by NSAIDS have been shown to be mediated partly through suppression of this Wnt pathway, resulting in the induction of *β*-catenin degradation [41]. Other potential therapeutics include small molecule inhibitors, such as lithium chloride which can modulate the Wnt signaling cascade, Wnt-blocking antibodies which have been shown to inhibit cell proliferation and induce apoptosis in a variety of cancer types, and Wnt-modulatory peptides which have been shown to decrease tumor growth and disrupt Wnt signaling [40].

*PIK3CA* mutations occur in about 10–20% of all ATC cases (Table 1) [38].* PIK3CA* encodes the p110α catalytic subunit of phosphoinositide 3-kinase, which functions to activate the PI3K/Akt/mTOR pathway to regulate cell cycle progression, adhesion, and motility [25]. Because of its ability to activate oncogenic activity,* PIK3CA* is of major interest for targeted drug therapy, as multiple alpha-specific oral PIK3CA inhibitors are available and are demonstrating responses in clinical trials [35].

In addition to the above, the oncogene* AKT1* is overexpressed and plays a key role in thyroid tumorigenesis [42, 43]. This activation occurs in approximately 5–10% of ATC cases (Table 1). While Akt activation promotes resistance to radiation therapy and standard chemotherapy, targeted therapeutics such as small-molecule inhibitors of the Akt serine/threonine kinases have the potential to inhibit Akt signals and can potentially induce apoptosis and decrease tumor cell growth in those cells which require the elevation in Akt signaling [44, 45].

Mutations in* BRAF* and* RAS* are commonly found in well-differentiated thyroid cancers, but they are also found in poorly differentiated and undifferentiated thyroid cancer types, so it would be reasonable to suggest that these mutations represent an early event in the progression of thyroid cancers [4]. It remains to be determined at which stage of thyroid cancer development activation of the other oncogenes discussed above occurs.

### 4.2. Tumor Suppressors

Mutations in the tumor suppressor* TP53* are commonly associated with 50–80% of ATC cases (Table 1). This gene, which encodes the cell cycle regulator p53, results in loss of this tumor suppressor expression and leads to impairment of the control of cell proliferation and apoptosis [4, 8]. There is great interest in targeting* TP53* because of the high frequency of* TP53* mutations in cancer; however no successful pharmacologic strategies to replace lost tumor suppressor function have been successful to date [8].

*PTEN* is a tumor suppressor gene and its mutated form is associated with 5–15% of ATC cases (Table 1). Through epigenetic effects, methylation of* PTEN* has been implicated in the progression of benign thyroid adenoma to aggressive ATC [25].* PTEN* methylation is associated with decreased, or complete lack of, PTEN expression and has also been found to stimulate other genetic alterations associated with the PI3K/Akt/mTOR pathway axis, including both* RAS* and* PIK3CA* [25]. While restoring PTEN tumor suppressing activity is much more difficult, PTEN loss is potentially druggable through targeting the downstream deregulated PI3K pathway with PI3K inhibitors [46].

### 4.3. Chromosomal Rearrangements

Structural alterations (e.g., copy number variation, translocations, and fusions) in addition to single nucleotide polymorphism (SNP) mutations can be determined by whole genome sequencing (WGS) in order to attempt to understand why some cancers such as ATC are more aggressive and less responsive to treatments than others. One explanation has proposed that chromosomal rearrangements, often identified more frequently in cancers that are more aggressive, may provide the answer. In particular, anaplastic lymphoma kinase (*ALK*) gene fusions have been associated with aggressive thyroid cancers, whereby rearrangements with* ALK* result in tumorigenic activity. One of the* ALK* rearrangements described involves the striatin (*STRN*) gene observed more commonly in thyroid cancers. This* STRN-ALK* fusion results in constitutive activity of ALK kinase and induction of thyroid cell proliferation and tumorigenesis [47]. In addition to* STRN-ALK* gene fusions, the echinoderm microtubule-associated protein-like 4 (*EML4*) gene has been reported as another gene fusion with* ALK* [48]. This fusion is more commonly described in lung cancer and results in constitutive ALK activation leading to cell proliferation and tumorigenesis [48].* EML4-ALK* has also been found to be a potent oncogenic activator in cell lines and genetically engineered mouse models [49]. Treatment with crizotinib, a small molecule ALK inhibitor, has demonstrated significant responses and improved disease-free survival in other cancers [50]. Additionally,* in vitro* studies in ATC cells harboring* ALK* fusions have demonstrated a 65% reduction in ATC proliferation compared to 20% for standard chemotherapy [47, 48].

In addition to the fusions described above, rearrangement of the peroxisome proliferator-activated receptors*γ* (*PPAR* *γ*) with the* PAX8* gene, associated with radiation induced thyroid cancers, expresses a fusion protein (PAX8/PPAR*γ*) [51]. This fusion has been found to accelerate cell growth, inhibiting normal tumor suppression by PPAR*γ* and therefore acts as an oncogene [51]. PPAR*γ* agonists such as troglitazone, rosiglitazone, and pioglitazone have been tested* in vitro* and exhibited growth inhibitions as well as apoptosis of tumor cells [51]. While results from various studies regarding PPAR*γ* agonists are promising, they still require more trials with larger groups and longer followups to be conclusive [51].

Other rearrangements common to thyroid cancers include* RET/PTC1* and* RET/PTC3*, which both result from intrachromosomal paracentric inversions and have a strong correlation with radiation exposure [4, 52]. The RET tyrosine kinase becomes activated by this rearrangement, leading to constitutively activated MAPK signaling and tumorigenesis in thyroid cells [4]. Sunitinib, a multitargeted receptor tyrosine kinase inhibitor, acts as an inhibitor of RET and exhibits some efficacy in these tumor types [53].

Chromosomal rearrangements involving neurotrophic tyrosine receptor kinases (*NTRK*s) can also occur in thyroid carcinomas and are known as TRK rearrangements.* NTRK1* and* NTRK3* fusions have been reported in multiple cancers including glioblastoma, lung cancer, and in 1-2% of PTC [21, 54, 55] and have a strong correlation with exposure to radiation [4, 56]. These fusions can involve multiple different partners leading to constitutive kinase activation [54, 55, 57]. Most importantly, small-molecule inhibitors of TRK are available and selectively inhibit the proliferation and colony formation of cell lines harboring TRK fusions but had no effect on wild type lines [55]. While TRK fusions have not yet been reported in ATC, it is likely that future NGS studies will reveal these genomic alterations similar to other mutations that are shared with well-differentiated cancers. Given the responses observed with other patients with ATC harboring other fusions, TRK inhibitors will likely be of clinical benefit to patients harboring NTRK fusions.

### 4.4. The Role of MicroRNAs

MicroRNAs (miRNAs) are small, single-stranded, noncoding RNAs, which are able to bind to gene targets and repress posttranscriptional gene expression [39]. They may act as oncogenes and/or tumor suppressors in the same cell, whereby inducing effects on cell differentiation, proliferation, and apoptosis leading to tumorigenesis [58, 59]. The specific miRNA expression profiles have been shown to significantly differ amongst medullary thyroid cancers when compared to cancers derived from follicular cells; however, amongst those derived from follicular cells, there are variable miRNA expression profiles [58]. Previous studies have shown that there is significantly reduced expression of miR-26a, miR-30a-5p, miR-30d, and miR-125b in ATC. Overexpression of miR-26a and miR-125b in two ATC human-derived cell lines resulted in cell growth inhibition, suggesting that these miRNAs are involved in cell cycle regulation [58]. Additionally, a group of seven miRNAs known as miR-17-92 (miR-17-3p, miR-17-5p, miR-18a, miR-19a, miR-19b, miR-20a, and miR-92-1) have been shown to be upregulated in ATC cell lines and lesions. While miRNAs are not directly druggable, their presence in the bloodstream or the tumor tissue has been useful for early detection or prognostic biomarkers [60]. They may be highly useful in both manners to improve the care of patients with ATC.

### 4.5. Gene Methylation

Gene methylation of promoter regions leading to interrupted transcription is commonly implicated in thyroid cancers [38]. Methylation of the promoter region of the* PTEN* gene leading to its silencing is commonly associated with ATC.* PTEN* methylation is frequently associated with the genetic alterations in the PI3K-AKT pathway in thyroid tumors, including mutations of* RAS* and* PIK3CA* mutation and amplification [38]. Lack of PTEN regulation in this setting creates a self-amplifying loop of PI3K-AKT pathway activation, reinforcing the importance of this axis in driving thyroid oncogenesis [38]. Further examination of PI3K-AKT pathway inhibition is warranted in ATC.

## 5. Conclusion

ATC, a devastating cancer resulting from genomic instability and a multitude of mutations, is universally fatal with no effective therapies. Survival rates have changed little over the past 30 years, which suggests that traditional methods of surgery, radiation, and chemotherapy are inadequate. Comprehensive genetic characterization of ATC tumors has the potential to identify effective novel agents, as well as biomarkers of drug sensitivity and resistance. New individualized therapeutics, based on genomic analysis of individual tumors, may hold the promise of effective treatment. These findings will likely have broader implications due to significant genetic overlap with well-differentiated thyroid cancer. Genomically derived treatment as part of a multidisciplinary approach may hold the promise of successful treatment for patients diagnosed with ATC.

## Figures and Tables

**Table 1:** Types of thyroid cancers, prevalence, and associated genetic profiles.

Characteristics	Papillary carcinoma	Follicular carcinoma	Poorly differentiated carcinoma	Anaplastic (undifferentiated) carcinoma	Medullary carcinoma

Cell type	Follicular	Follicular	Follicular	Follicular	C cell
Prevalence (%)	80–85	10–15	<2	1-2	3–5
Typical route of spread	Local lymph-node metastasis	Hematogenous metastasis, typical to bones and lungs	Invasive local growth, lymph-node, and hematogenous metastases	Invasive local growth, lymph-node and hematogenous metastases	Lymph-node and hematogenous metastases
10-year survival (%)	95–98	90–95	~50	<10	60–80
Common mutations and prevalence (%)	*BRAF* 40–45 *RAS* 10–20 *RET/PTC* 10–20 *TRK* < 5	*RAS* 40–50 *PAX8/PPAR* *γ* 30–35 *PIK3CA* < 10 *PTEN* < 10	*RAS* 20–40 ***TP53***∗*** 20–30*** *BRAF* 10–20 ***CTNNB1***∗*** 5–10*** *PIK3CA* 5–10 ***AKT1***∗*** 5–10***	***TP53*** ∗ *** 50–80*** ***CTNNB1***∗*** 5–60*** *RAS* 20–40 *BRAF* 20–40 *PIK3CA* 10–20 *PTEN* 5–15 ***AKT1***∗*** 5–10***	Familial forms: *RET* > 95 Sporadic: *RET* 40–50 *RAS* 25

*Gene mutations unique to poorly differentiated and undifferentiated (anaplastic) thyroid carcinomas.

Adapted from Nikiforov and Nikiforova 2011 [4].
